# Modulation of Urate Transport by Drugs

**DOI:** 10.3390/pharmaceutics13060899

**Published:** 2021-06-17

**Authors:** Péter Tátrai, Franciska Erdő, Gabriella Dörnyei, Péter Krajcsi

**Affiliations:** 1 Solvo Biotechnology, Science Park, Building B2, 4-20 Irinyi József utca, H-1117 Budapest, Hungary; peter.tatrai@crl.com; 2 Faculty of Information Technology and Bionics, Pázmány Péter Catholic University, H-1083 Budapest, Hungary; erdo.franciska@itk.ppke.hu; 3 Department of Morphology and Physiology, Faculty of Health Sciences, Semmelweis University, H-1088 Budapest, Hungary; dornyeig@se-etk.hu

**Keywords:** urate, drug-transporter interactions, in vitro prediction, hyperuricemia, hypouricemia

## Abstract

Background: Serum urate (SU) levels in primates are extraordinarily high among mammals. Urate is a Janus-faced molecule that acts physiologically as a protective antioxidant but provokes inflammation and gout when it precipitates at high concentrations. Transporters play crucial roles in urate disposition, and drugs that interact with urate transporters either by intention or by accident may modulate SU levels. We examined whether in vitro transporter interaction studies may clarify and predict such effects. Methods: Transporter interaction profiles of clinically proven urate-lowering (uricosuric) and hyperuricemic drugs were compiled from the literature, and the predictive value of in vitro-derived cut-offs like C_max_/IC_50_ on the in vivo outcome (clinically relevant decrease or increase of SU) was assessed. Results: Interaction with the major reabsorptive urate transporter URAT1 appears to be dominant over interactions with secretory transporters in determining the net effect of a drug on SU levels. In vitro inhibition interpreted using the recommended cut-offs is useful at predicting the clinical outcome. Conclusions: In vitro safety assessments regarding urate transport should be done early in drug development to identify candidates at risk of causing major imbalances. Attention should be paid both to the inhibition of secretory transporters and inhibition or trans-stimulation of reabsorptive transporters, especially URAT1.

## 1. Urate Homeostasis and Its Disorders

**Figure 1:**
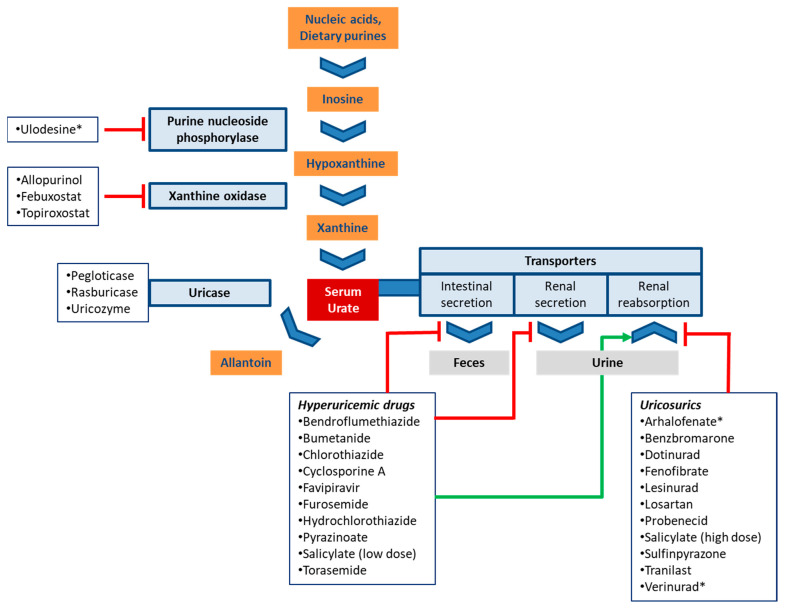
Schematic routes of urate production and disposition. The boxes list drugs that affect urate homeostasis and are discussed in this review. Experimental drugs are marked with an asterisk (*). Red bars (inhibition) and the green arrow (stimulation) indicate the main mechanism by which drugs modulate serum urate levels.

Urate is the end product of purine catabolism in primates including humans. In other mammals, urate is broken down to allantoin by the enzyme uricase (urate oxidase). This function, however, was lost in the evolutionary lineage of primates due to a mutation [1]. Purines are derived from dietary sources, the breakdown of nucleic acids, as well as from de novo synthesis. Urate is synthesized in the liver and the small intestine [2]. The last two steps of urate synthesis are catalyzed by xanthine oxidase (XO) that converts hypoxanthine to xanthine and xanthine to uric acid (Figure 1) [2].

Approximately two-thirds of urate is excreted renally and one-third with feces [3]. About 15% and 10% of plasma urate is protein-bound in males and females, respectively [4]. In healthy adults the average fractional excretion of uric acid (FE = fraction secreted/fraction filtered) is 6–8% [5]. After glomerular filtration, 90–97% of urate is reabsorbed in the proximal tubule, and tubular secretion of urate also takes place. However, the exact location and temporal sequence of reabsorption and secretion is as yet unclear [5].

Hyperuricemia is a condition characterized by elevated serum urate (SU) levels, and is defined as SU concentration exceeding 357 μmol/L (6.0 mg/dL) in women and 416 μmol/L (7.0 mg/dL) in men [6,7,8]. Hyperuricemia results from an imbalance between the production and excretion of urate, and can be classified as (i) renal underexcretion type (RUE) where FE is ≤5.5% and urinary urate excretion (UUE) is ≤25%; (ii) renal overload (ROL) type when FE ≥5.5% and UUE is ≥25%; (iii) combined type when FE is ≤5.5% and UUE is ≥25%; and (iv) normal type when FE ≥ 5.5 and UUE is ≤25%. Gout, one of the most frequent diseases caused by hyperuricemia, can also be categorized accordingly [9]. Overproduction of urate is one of the main underlying causes of hyperuricemia, while dietary factors such as consumption of protein-rich food may also contribute to the elevation of SU [10].

Hyperuricemia may lead to kidney stones, urate nephropathy, or even kidney failure [11]. While hyperuricemia undoubtedly increases the risk of gout, the two conditions cannot be equated, as hyperuricemia is by far more prevalent than gout [12]. Hyperuricemia is also a risk factor for cardiovascular disease [13]: a significant increase in the hazard ratio for cardiovascular mortality was observed with SU levels ≥ 7 mg/dL in men and ≥5 mg/dL in women, and a similar trend was seen in all-cause mortality [14]. More recently, hyperuricemia has been considered as an indicator of metabolic syndrome and diabetes [15].

Hypouricemia, recently reviewed in [16], is a condition characterized by SU levels lower than 2 mg/mL (119 µM). Hypouricemia is traditionally considered a biochemical curiosity with low clinical significance [16] and clinical guidance on renal hypouricemia has been updated only recently [17]. Hereditary hypouricemia is most commonly associated with mutations in the SLC22A12/URAT1 and the SLC2A9 / GLUT9 genes [18,19]. Exercise-induced acute kidney failure may develop in patients with URAT1 or GLUT9 mutations due to oxygen radical-induced spasms of the renal vasculature [20,21]. Drug-induced hypouricemia is rare and has not been studied as extensively as drug-induced hyperuricemia. Whenever observed, drug-induced hypouricemia mostly occurs as an overshoot of urate-lowering therapies [16]. Albeit rare in clinical practice, manifestations of hypouricemia may include kidney injury and neurological syndromes. Hence, it is necessary to assess drug liabilities with regard to both the secretory and reabsorptive arms of urate transport.

Transporter gene polymorphisms associated with disturbed urate homeostasis have been reported (known associations between mutations and SU levels are marked in Table 1) and recently reviewed in Wang et al. [22]. Therefore, pharmacogenetic testing may reveal important clues about the pathogenesis of urate imbalance, and heterologous expression of mutant transporters in cells could shed light on the effect of polymorphisms on transmembrane transport. Polymorphisms may not only affect transport properties but also the stability or trafficking of the protein encoded. Polymorphisms affecting transport without interfering with stability or trafficking are more likely to result in differential response of the transporter to drugs, although studies to clarify this are scarce.

## 2. The Double Face of Urate

Urate is an endogenous antioxidant that reduces lipid peroxidation and decreases the amount of nitric oxides via chemical scavenging or inhibition of their production [23]. Urate as a potent iron chelator can also minimize iron-mediated redox reactions and the generation of reactive oxygen species (ROS) [24]. These beneficial effects substantiate the potential evolutionary benefit of maintaining high systemic levels of urate. This metabolic strategy, however, turned out to be a double-edged sword, because the antioxidant activity of urate plateaus at concentrations exceeding its maximal solubility (6–7 mg/dL), where monosodium urate crystals form [25]. In macrophages, monosodium urate crystals activate intracellular pattern recognition receptors such as NLRP3, and probably also Toll-like receptors (TLR) on the cell surface. These interactions trigger the assembly of inflammasomes and activation of the caspase pathway, precipitating cell death by pyroptosis. The mechanism of pyroptosis involves formation of pore-like structures in the plasma membrane and the release of inflammatory cytokines (e.g., IL-1β) [25]. Propagation of the inflammatory response may lead to inflammatory arthritis and chronic gout.

High urate levels have also been linked to blood pressure [26]. Growing evidence indicates that asymptomatic hyperuricemia is involved in the development of hypertension via activation of the renin–angiotensin system and inhibition of nitric oxide synthesis, which promotes endothelial dysfunction, proliferation of vascular smooth muscle cells, and sodium reabsorption [27,28]. Deposition of urate crystals (extracellular uric acid) in the urinary lumen and in the endothelium of arteries can cause inflammation and pro-inflammatory responses, respectively [28]. It has also been demonstrated that high levels of intracellular uric acid can upregulate the aldose reductase enzyme and induce mitochondrial dysfunction and superoxide generation. All these mechanisms seem to contribute to the development of endothelial damage and thus to the pathogenesis of hypertension. A pilot study monitoring blood pressure of adolescents with stage 1 primary hypertension and borderline hyperuricemia demonstrated that blood pressure decreased upon administration of allopurinol, a conventional antihyperuricemic medication [29]. The study, however, had limitations as detailed in the article, and a more recent review that acknowledges serum urate level as an independent risk factor for hypertension urges further studies to explore if a reduction of serum urate could be beneficial in preventing or treating hypertension [27,30].

## 3. Transporters of Urate

**Figure 2:**
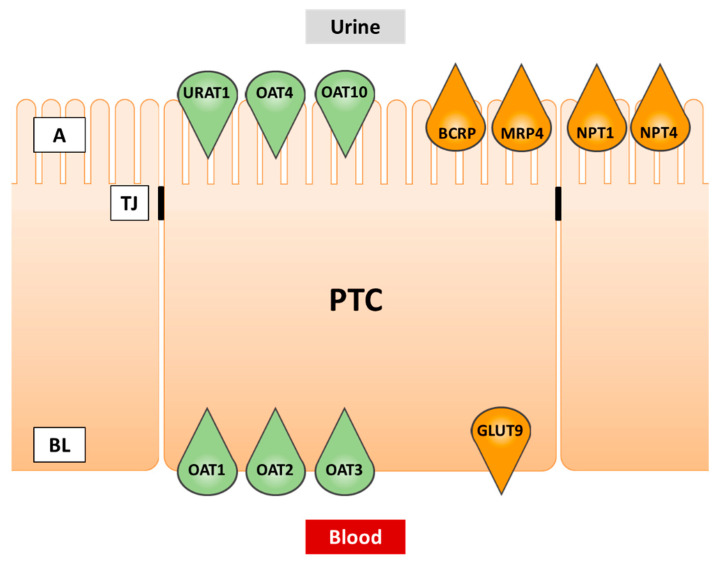
Transporters of urate in renal proximal tubule cells. Green fill color: transporters involved in cellular uptake. Orange fill color: transporters involved in cellular efflux. PTC, proximal tubule cell; A, apical (urine) side; BL, basolateral (blood) side; TJ, tight junction.

The main sites of urate transport are the kidney and the intestine [31]. Although multiple urate transporters have been detected in the intestine [32], a detailed characterization from a drug inhibition point of view is available for BCRP only, and the key urate transporter URAT1 is not significantly expressed in the gut. In the kidney, on the other hand, numerous transporters including URAT1 cooperate in urate disposition, and the kidney is the principal target of SU-modulating therapies. Therefore, renal urate transport will be covered in more detail in this review. Transporters expressed in proximal tubule cells and involved in urate transport are summarized in Table 1 and depicted in Figure 2.

### 3.1. Secretory Renal Urate Transporters

#### 3.1.1. OAT1/*SLC22A6* and OAT3/*SLC22A8*

OAT1 and OAT3 are highly expressed in the basolateral membrane of kidney proximal tubule cells (PTC) and are generally thought to be responsible for organic anion uptake; thus, both are important contributors to the tubular secretion of anions [33]. Both human OAT1 [34] and OAT3 [35] transport urate, and significantly lower levels of urate were detected in the urine of Oat1- and Oat3-knockout mice [36]. The human OAT1 variant rs148838714 was shown to be significantly associated with serum urate levels, although exactly how this variation affects urate transport is not yet known [37]. When comparing OAT1-mediated tenofovir transport across orthologs of different mammalian species including primates and non-primates, the efficiency of transport was found to correlate with serum urate levels, suggesting that evolutionary changes in primates likely reflect adaptation to an increased urate load [38].

#### 3.1.2. OAT2

Compared to OAT1/3, OAT2 displays different substrate specificity and a broader tissue expression profile; nevertheless, it also efficiently transports urate [39]. Since it is expressed at a lower level relative to OAT1 and OAT3 in the kidney [40], and is generally thought to be less prone to drug-mediated inhibition, the effect of uricosuric agents on OAT2 has not been extensively characterized, but OAT2-mediated inhibition may be responsible for the hyperuricemic effect of bempedoic acid [41,42].

#### 3.1.3. BCRP/*ABCG2*

BCRP is expressed in the apical membrane of all major physiological barriers. It is a low affinity, high-capacity urate transporter [43] and is thought to play a key role in secretory urate transport in both the kidney [26] and the intestine [31]. BCRP expressed in the canalicular membrane of liver cells may also play a minor role in the excretion of hepatically synthetized urate into the bile [44]. *Abcg2*/Bcrp1 knockout mice showed increased plasma levels of urate [3], and multiple GWAS studies demonstrated associations between human allelic variants with impaired urate transport in vitro and hyperuricemia [26]. A recent study concluded that mutations in BCRP cause gout and these mutations occur with significant prevalence in the general population [45].

#### 3.1.4. MRP4/*ABCC4*

MRP4 is expressed in the luminal membrane of PTCs [46] and facilitates the efflux of urate in vitro [47]. MRP4 is also expressed in the sinusoidal membrane of hepatocytes and was shown to contribute to the secretion of hepatically synthetized urate into the blood [44]. A recent population-specific resequencing identified an MRP4 variant, rs4148500, which was significantly associated with hyperuricemia and gout. This rare allele encodes a P1036L mutation and showed a 30% decrease in urate transport when expressed in *Xenopus* oocytes [48].

#### 3.1.5. NPT1/*SLC17A1* and NPT4/*SLC17A3*

NPT1 and NPT4 are expressed in the apical membrane of PTCs [49]. NPT1, originally identified as phosphate transporter, is a Cl^−^-dependent anion effluxer involved in urate excretion under physiological conditions [50]. NPT4 is a voltage-driven urate transporter [51]. Variants of both transporters that were shown to display impaired transport of urate in vitro were linked to hyperuricemia and gout in GWAS studies [26]. Notably, a gain-of-function variant of NPT1 rs1165196 (I269T) with increased urate transport activity [52] was found to decrease the risk of underexcretion type gout [53].

### 3.2. Reabsorptive Renal Urate Transporters

#### 3.2.1. URAT1/*SLC22A12*

URAT1 is localized in the apical membrane of PTCs [18]. URAT1 is the centerpiece of urate physiology and loss-of-function mutations cause idiopathic renal hypouricemia [16] with serum urate levels as low as ≤2.0 mg/dL [16]. In Japan, 80–90% of hereditary renal hypouricemia patients are homozygous or compound heterozygous for mutations in the gene of hURAT1 [54,55]. In Urat1-knockout mice the urate/creatinine ratio was decreased in the urine, whereas no significant change in serum urate levels were observed [56], indicating that transporters other than Urat1 may play a role in urate reabsorption in mice.

#### 3.2.2. OAT4/*SLC22A11* and OAT10/*SLC22A13*

Both OAT4 and OAT10 are localized in the apical membrane of PTCs [57]. OAT4 [58] and OAT10 [59] are thought to transport urate in exchange for OH^−^ ions. The OAT4 variant rs17300741 was associated with renal underexcretion type gout [60], and carriers of a missense variant (R377C) of OAT10 that was non-functional in urate transport in vitro showed reduced SU levels and decreased susceptibility to gout [61].

#### 3.2.3. GLUT9/URATv1/*SLC2A9*

GLUT9 has two isoforms: GLUT9a and GLUT9b. Both mediate urate transport utilizing glucose and fructose as exchange substrates [62]. GLUT9a mediates reabsorption at the basolateral membrane, while GLUT9b in an apical position may operate in both directions [63]. Early observations showed that missense mutations in GLUT9 led to hypouricemia [64]. Several subsequent GWAS studies linked mutations in GLUT9 to hypouricemia [65]. Genetic ablation of Glut9 in mice leads to moderate hyperuricemia and increased renal excretion of urate. Defects in the conversion of urate into allantoin and defective renal reabsorption of urate were proposed as potential mechanisms [23]. In addition, as Glut9 is also expressed in the gut and the liver, and a study on sandwich-cultured human hepatocytes suggested a role for GLUT9 in sinusoidal efflux of urate [44], genetic ablation of Glut9 may interfere with the intestinal excretion of urate [66].

## 4. In Vitro Methods to Investigate the Interaction of Urate Transporters with Drugs

The effect of drugs on the transport of urate, whether it is the intended pharmacodynamic action or an undesired liability, can be investigated in in vitro model systems, and results obtained from these models can help to predict clinical response by extrapolation to the in vivo setting.

In in vitro transporter assays, transporter-dependent accumulation or vectorial translocation of a probe substrate is quantified using an appropriate method. The mode of detection depends on the probe substrate used and may be spectrophotometry for colored substrates, fluorometry or flow cytometry for fluorescent substrates, liquid scintillation counting for radiolabeled substrates, or quantitative mass spectrometry for unlabeled substrates. In transporter interaction assays, a test drug is added at a single dose or multiple doses, and the effect of the drug on probe substrate transport—which can be inhibitory or stimulatory—is determined. Test drugs can be added in cis, i.e., on the same side, or in trans, i.e., on the opposite side of the membrane relative to the probe substrate. While inhibition studies can be performed in either the cis or the trans setup, they are commonly done in the cis setup, whereas the trans configuration is more accustomed in stimulation studies.

### 4.1. Cellular Expression Systems

In vitro transporter assays require cell lines that express the transporter of interest at a high level. Some cell lines like Caco-2, a human colon cancer cell line with small intestine-like differentiation, display sizable intrinsic expression of multiple pharmacologically relevant transporters including BCRP, and may thus be used for transporter studies without further modification (e.g., [67]). More often, however, the transporter of interest is introduced exogenously into a host cell, preferably one with low intrinsic expression of transporters. The *Xenopus* oocyte system has been used since the early days of transporter studies. Heterologous expression of transporters in the *Xenopus* oocyte is achieved by microinjection of complementary RNA (e.g., [68]). Immortalized cell lines like Chinese Hamster Ovary (CHO), Human Embryonic Kidney-293 (HEK-293), Madin-Darby Canine Kidney II (MDCKII) derived from dog renal proximal tubules, or S2 derived from mouse renal proximal tubules can be transfected transiently or stably, or transduced using viral vectors to achieve high functional expression of mammalian transporters (e.g., [34,69,70]). The same overexpressing cells, as well as baculovirus-transduced *Sf9* insect cells, may also be processed to prepare membrane vesicles for vesicular transport assays (see below) (e.g., [71,72,73]). Insect cell membranes are sometimes preferred for their superior signal-to-noise ratio due to high expression of the transporter of interest against low background transport activity.

### 4.2. Assay Types

ATP hydrolysis-driven export pumps of the ATP-binding cassette (ABC) family and facilitative or secondarily active transporters of the solute carrier (SLC) family are assayed by different methodologies. Current methods in widespread use for interaction studies with ABC transporters include the vesicular transport assay, the vectorial transport or monolayer assay, and the substrate efflux assay, while traditional ATPase assays are becoming obsolete. Vesicular transport assays utilize inside-out membrane vesicles prepared from cells overexpressing the transporter of interest. Such inverted membrane vesicles contain the transporter in a reverse orientation and therefore pump substrates in an inward direction. The assay endpoint is ATP-dependent accumulation of the probe substrate. In vectorial transport or monolayer assays, cells forming a tight monolayer like MDCKII (e.g., [73]) are grown on a transwell membrane support that separates a basolateral and an apical fluid compartment. Transcellular flux of the probe substrate in both apical-to-basolateral (A-to-B) and basolateral-to-apical (B-to-A) directions is monitored over time, and the apparent permeability (P_app_) for each direction is calculated. For apically located efflux transporters like BCRP, the efflux ratio (ER) is defined as the ratio of P_app, B-to-A_ to P_app, A-to-B_, where A-to-B flux represents passive transport minus oppositely directed active transport, while B-to-A flux represents passive plus active transport. Interaction of a drug with the transporter is assessed by its effect on the ER. In substrate efflux assays, transporter activity is quantified based on the exclusion of a probe substrate from cells. For example, the extrusion of pheophorbide A from MDCKII-hBCRP cells was measured by flow cytometry [74], and efflux of pre-loaded ^14^C-urate from HEK293-MRP4 cells was quantified by liquid scintillation [71]. In these assays, the externally administered test drug is acting in trans with respect to the direction of transport.

In drug interaction studies with SLC transporters, transfected/transduced cell lines or cRNA-injected *Xenopus* oocytes are typically exposed to the probe substrate and the test drug in cis, and the effect of the drug on cellular uptake of the substrate is assessed. Alternatively, in the case of bidirectional (exchange) transporters such as URAT1 or OATs, the substrate may be pre-loaded into the cells by microinjection or pre-incubation, and the test drug is added externally (i.e., in trans) while the pre-loaded substrate is being effluxed. For example, non-steroidal anti-inflammatory drugs (NSAIDs) were found to trans-stimulate the efflux of pre-loaded ^14^C-labeled urate from human OAT1-expressing oocytes [75].

It is important to note that probe substrates other than urate were used extensively in the studies referenced in this review. Although all transporters discussed herein contribute significantly to urate disposition, many of them—especially OATs, BCRP, and MRP4—have other important physiological and pharmacological substrates which are used preferentially as probes in in vitro drug interaction assays (see Table 1). In fact, owing to its poor aqueous solubility, urate is not a particularly convenient in vitro substrate to work with. Although the interaction between a drug and a substrate may depend on the nature of the latter, and thus results obtained with one substrate may not be readily transferable to others, a wealth of relevant information would be excluded by rejecting transporter interaction data obtained with substrates other than urate.

## 5. Therapeutic Approaches to Treat Hyperuricemia and Gout

Treatment of hyperuricemia may target the metabolism of urate via inhibition of urate production or facilitation of its breakdown. This approach is briefly discussed in 5.1, but otherwise not covered herein. Instead, the present manuscript focuses on another therapeutic strategy, the inhibition of transporters involved in urate reabsorption [76]. Both approaches are depicted in Figure 1 with select drugs and drug candidates.

Urate-lowering therapies must be administered with caution as accidentally overtreated hyperuricemia may give way to the opposite condition, hypouricemia [16]. The emergence of URAT1 inhibitory drugs provided the first insights into this arm of urate homeostasis and transport, and hypouricemia was reported upon application of uricosuric agents.

### 5.1. Approaches Targeting the Metabolism of Urate

Current therapeutic options to reduce serum urate levels include inhibition of urate production by xanthine oxidase and administration of recombinant uricase [77]. The lack of uricase, an evolutionary trait humans share with all primates, is the major metabolic factor behind universally high SU levels in our species. Uricase-deficient rats named “Kunming-DY” rats have been engineered using the CRISPR/Cas9 technique. Kunming rats were healthy with more than 95% survival up to one year. Mean SU in male Kunming rats was 48.3 µg/mL, significantly higher compared to wild-type male Sprague-Dawley rats (under 20 µg/mL in the study) [78]. In the rat model of fructose-induced hyperuricemia, treatment with uricase-expressing engineered bacteria significantly decreased serum urate levels [79], supporting the notion that exogenous uricase may improve hyperuricemia.

The first uricase to enter clinical practice, uricozyme, has been indicated for tumor lysis syndrome-associated hyperuricemia [80], but it occasionally precipitates a hypersensitivity reaction mainly manifesting in bronchospasm [81]. Rasburicase, a recombinant uricase also used to treat patients with tumor lysis syndrome-associated hyperuricemia, shows better specific activity, which is thought to be due to the lower likelihood of adduct formation, a purification problem that complicates the manufacturing process of uricozyme [82]. Rasburicase, however, was found to induce the production of anti-uricase antibodies in 11–64% of patients [83]. To suppress antibody formation against recombinant uricase, a PEGylated formulation of porcine uricase, pegloticase, was developed. Pegloticase was efficient at reducing serum urate levels in patients with refractory gout, but a portion of patients developed anti-PEG antibodies against pegloticase and showed adverse reactions [84].

Allopurinol, the prototype inhibitor of xanthine oxidase, has been used for decades in the treatment of hyperuricemia. Newer members of the xanthine oxidase inhibitor family include febuxostat and the most recent topiroxostat. In a meta-analysis of randomized controlled trials, 120 mg/day febuxostat showed superior efficacy compared to 200 mg/day allopurinol or 120/160 mg/day topiroxostat, while topiroxostat and allopurinol had fewer associated adverse events [85].

Mechanistically, urate production can be reduced at an even earlier step by inhibiting purine nucleosidase phosphorylase (PNP), the enzyme that catalyzes conversion of inosine to hypoxanthine. Hypoxanthine is the precursor of xanthine and a substrate of xanthine oxidase (Figure 1). Ulodesine, a PNP inhibitor, was effective as single agent [86] and in combination with allopurinol [87]. In the combination treatment arm, 40–55% of patients achieved the target urate level, which compared favorably to 25% in the allopurinol arm [88].

### 5.2. Approaches Targeting the Reabsorption of Urate: Uricosuric Agents

Uricosuric agents are defined as drugs that shift the balance of urate disposition towards net secretion, thereby decreasing serum urate levels. Pharmacokinetic properties of uricosurics discussed in this review are listed in Table 2. Uricosurics are thought to exert their action predominantly via inhibition of URAT1, the central player in the renal reabsorption of urate, and therefore a primary target of these drugs; however, in in vitro investigations many of them have turned out to inhibit multiple urate transporters, both intestinal and renal (see Table 3). While it is easy to see how inhibiting other reabsorptive transporters such as OAT4 or OAT10 may contribute to the uricosuric action of a drug, a more complex picture emerges when secretory-type transporters like OAT1, OAT3, or BCRP are inhibited concomitantly with URAT1. In such cases—which appear to be more the rule than the exception—the net outcome is dictated by the relative inhibitory potencies of the same drug against various synergistic and antagonistic transporters (see Table 4).

Albeit some old uricosurics like benzbromarone or probenecid have persisted on the market for decades, the discovery of novel uricosuric drugs is still a hot field with many candidate drugs in various phases of development [76,89,90]. Nevertheless, in this review we only discuss drugs and drug candidates with a clinically confirmed uricosuric effect and a well-characterized and publicly available transporter interaction profile. For each drug–transporter pair, the potential clinical significance of interaction was assessed based on the ratio of gut/plasma concentration and in vitro measured IC50 or Ki according to the following cut-off values: (i) intestinal BCRP: R = 1 + C_gut_/(IC_50_ or K_i_) ≥ 11; (ii) renal transporters: C_max,u_/(IC_50_ or K_i_) ≥ 0.02 or ≥ 0.1 (both cut-offs have been used by major regulatory agencies) (Table 3). Table 4 shows inhibition potencies relative to URAT1, i.e., the IC50 of each drug for a given transporter was compared to the IC50 of the same drug for URAT1. IC50 ratios > 1 indicate weaker inhibition, and IC50 ratios < 1 indicate stronger inhibition of a given transporter compared to URAT1.

**Table 2:** Pharmacokinetic properties of uricosurics and hyperuricemic drugs discussed in this review. N/A, not available. ^1^ Assuming f_u_ = 0.01 (if f_u_ is not available, or the measured f_u_ is <0.01). ^2^ Pharmacokinetic data from cynomolgus monkey. ^3^ Refers to the parent compound. ^4^ Steady-state trough concentration.

Drug/Compound	MW	Dose	C_gut_	C_max_	f_u_	C_max,u_	References
Uricosurics							
Benzbromarone	424	100 mg	943 µM	7 µM	0.01	0.07 µM	[91]
Probenecid	285	2000 mg	28.1 mM	520.7 µM	0.09	46.9 µM	[70,92,93]
Lesinurad	404	200 mg	1.98 mM	29 µM	0.016	0.46 µM	[94,95]
Verinurad	348	10 mg	115 µM	0.46 µM	0.02	0.0092 µM	[96]
Dotinurad	358	4 mg	44.8 µM	1.2 µM	0.007	0.012 µM ^1^	[69]
Arhalofenate	416	600 mg	5.77 mM	337 µM	N/A	3.37 µM ^1^	[97]
Fenofibrate	361	67 mg	742 µM	25.8 µM	0.01	0.236 µM	[98]
Fenofibric acid	319	-	-	N/A	N/A	0.81 µM	[99]
Tranilast	327	200 mg	2.45 mM	129 µM	N/A	1.29 µM ^1^	[100]
Losartan	423	50 mg	473 µM	0.60 µM	0.013	0.0078 µM	[101]
Sulfinpyrazone	405	200 mg	1.98 mM	48.1 µM	0.017	0.82 µM	[102]
Salicylate (high dose)	160	5200 mg	130 mM	1100 µM	0.25	275 µM	[103,104]
Epaminurad (UR-1102, URC-102) ^2^	414	~70 mg	676 µM	0.22 µM	N/A	0.0022 µM ^1^	[105]
Hyperuricemic drugs							
Bumetanide	364	1 mg	11.0 µM	0.8 µM	0.125	0.1 µM	[72,106,107]
Furosemide	331	80 mg	970 µM	8–17 µM	0.041	0.697 µM	[51,108]
Torasemide	348	200 mg	2.30 mM	~51.7 µM	0.01	0.517 µM	[109,110]
Chlorothiazide	296	1000 mg	13.51 mM	120–240 µM	>0.1	>24 µM	[51]
Hydrochlorothiazide	298	100 mg	1342 µM	1.64 µM	0.33	0.54 µM	[111]
Bendroflumethiazide	421	5 mg	47.5 µM	0.08–0.2 µM	0.05	4–10 nM	[112,113]
Salicylate (low dose)	160	1000 mg	25 mM	~280 µM	0.25	~70 µM	[103,104]
Pyrazinoate (pyrazinamide metabolite)	124	3000 mg ^3^	-	≤150 µM	0.69	≤103.5 µM	[34,114]
Cyclosporine A	1202	300 mg	1.66 mM	1.5 µM	0.122	0.183 µM	[115]
Favipiravir	157	2400 mg	61.1 mM	294 µM ^4^	0.46	135 µM ^4^	[116]
Favipiravir M1	173	1200 mg ^3^	-	87.9 µM	0.712	62.6 µM	[117]

**Table 3:** Inhibition of urate transporters by drugs with a clinically observed uricosuric effect. Interactions were predicted to be clinically significant based on the following cut-off values: for intestinal BCRP, R = 1 + C_gut_/(IC_50_ or K_i_) ≥ 11 (shown in **bold**); for renal transporters, C_max,u_/(IC_50_ or K_i_) ≥ 0.02 (shown in **bold** only) or ≥0.1 (shown in **bold and underlined**). Values in red were obtained using urate as a probe substrate. For each drug/transporter pair, results from different studies are separated by semicolons. All values are in µM. Abbreviations: Secr., secretion; Reabs., reabsorption; N/I, no inhibition; (H)/(L), high/low affinity transport. ^1^ Fenofibrate prodrug is present in the gut only; the effective species in the kidney is fenofibric acid.

Transporter	BCRP (Gut)	OAT1	OAT3	BCRP (Kidney)	MRP4	NPT1	NPT4	URAT1	OAT4	OAT10	GLUT9	
Role	Secr.	Secr.	Secr.	Secr.	Secr.	Secr.	Secr.	Reabs.	Reabs.	Reabs.	Reabs.	
Drug/Compound												References
**Benzbromarone**	IC50 = **0.289**	Ki = **0.22**; IC50 = 12.75; 13.23; **3.14**	Ki = **0.11**; IC50 = **0.967**	IC50 = **0.289**	**IC50** = **0.104****(H)**/15.6 (L)	IC50: 17.1		Ki = **0.052**; IC50 =**0.18**;**0.29**	**IC50** = **3.19**	IC50 > 3	IC50 ~ 100	[68,69,71,94,105,118,119]
**Probenecid**	**IC50** = **433**	IC50 = **49.68**; **4.66**; **10.9**	IC50 = **27.9**; **2.37**		IC50 = **132** (potentia-tion)			IC50 = **66.82**; **13.23****, 165**	IC50 = **15.54**			[69,70,71,94,118]
**Lesinurad**	IC50 > 3000; **26.4**; >100	IC50 = 43.99; **4.3**; **6.99**	IC50 = **1.07**; **3.5**	IC50 > 3000; 26.4; >100				IC50 = 65.47; **3.53**	IC50 = **2.03**		IC50 > 100	[69,94,95,118]
**Verinurad**		IC50 = 4.6						IC50 = **0.025**	IC50 = 5.9			[120]
**Dotinurad**	IC50 = **4.16**	IC50 = 4.08	IC50 = 1.32	IC50 = 4.16				IC50 = **0.0372**				[69]
**Arhalofenate**								IC50 = **92**	IC50 = **2.6**	IC50 = **53**		[119]
**Fenofibrate/Fenofibric acid ^1^**	IC50 = 170		Ki = **2.2**					IC50 = **35.68**				[98,121,122]
**Tranilast**	N/I	complete inh at 100	IC50 = ~**15**	N/I		IC50 = **18.9**		IC50 = ~**21**; Ki = **21.33**	IC50 = ~**22**	IC50 = ~**31**	(GLUT9a) IC50 = **15.6**; Ki = **17.13**	[68]
**Losartan**	N/I	IC50 = 12	IC50 = 1.6	N/I	IC50 = 1.5			Ki = **0.0077**	IC50 = 18		IC50 = ~1000 (mSlc2a9)	[74,101,123]
**Sulfinpyrazone**					IC50 = **0.16****(H)**/40 (L)			IC50 = 716; **3.4**				[71,120,124]
**Salicylate (high dose)**		IC50 = **1573.4**; Ki = **341**			IC50 = **2.1** **(H)/1547 (L)**			IC50 = **106**; **23.9**				[34,71,75,104,125]
**Epaminurad**		Ki = 7.2	Ki = 2.4					Ki = 0.057				[105]

**Table 4:** Relative inhibition potencies. The IC50 of each drug for a given transporter is compared with its IC50 for URAT1. For this calculation, the estimate IC50 = 2 × Ki was used when the IC50 was not available or 2 × Ki was lower than the available IC50. Values shown for each transporter are IC50_transporter_ followed by the ratio IC50_transporter_/IC50_URAT1_). Relative inhibition values >1 indicate weaker inhibition, values <1 indicate stronger inhibition compared to URAT1. The rules for highlighting in **bold** or **bold and underlined** are the same as in Table 3. Abbreviations: Secr., secretion; Reabs., reabsorption.

Transporter	OAT1	OAT3	BCRP (Kidney)	MRP4	NPT1	NPT4	URAT1	OAT4	OAT10	GLUT9	
Role	Secr.	Secr.	Secr.	Secr.	Secr.	Secr.	Reabs.	Reabs.	Reabs.	Reabs.	
Drug/Compound											References
**Benzbromarone**	**0.44 µM**/4.2	**0.22 µM**/2.1	**0.289 µM**/2.8	**0.104 µM**/1.0			**0.104 µM**/1	**3.19 µM**/30.7	>3 µM/>28.8	~100 µM/~961	[69,71,94,105,119]
**Probenecid**	**4.66 µM**/0.35	**27.9 µM**/2.11		**132 µM**/10.0			**13.23 µM**/1	**15.54 µM**/1.17			[70,71,94]
**Lesinurad**	**4.3 µM**/1.22	**1.07 µM**/0.31	26.4 µM/7.54				**3.53 µM**/1	**2.03 µM**/0.58		~100 µM/~28.6	[69,94,95]
**Verinurad**	4.6 µM/184						**0.025 µM**/1	5.9 µM/236			[120]
**Dotinurad**	4.08 µM/110	1.32 µM/35.5					**0.0372 µM**/1				[69]
**Arhalofenate**							**92 µM/1**	**2.6 µM**/0.028	**53 µM**/0.58		[119]
**Fenofibric acid**		**4.4 µM**/0.12					**35.68 µM**/1				[121,122]
**Tranilast**		**~15 µM**/0.71			**18.9 µM**/0.90		**~21 µM [68]**/1	**~22 µM**/1.05	**~31 µM**/1.48	**15.6 µM**/0.75	[68]
**Losartan**	12 µM/779	1.6 µM/104		1.5 µM/97.4			**0.0154 µM**/1	18 µM/1169			[101]
**Sulfinpyrazone**				IC50 = **0.16 µM** (H)/0.047; **40 µM** (L)/11.8			**3.4 µM**/1				[71,120,124]
**Salicylate (high dose)**	**682 µM**/14.3			**2.1 µM**/0.088			**23.9 µM**/1				[71,75,125]
**Epaminurad**	14.4 µM/126	4.8 µM/42.1					0.114 µM/1				[105]

#### 5.2.1. Benzbromarone

Benzbromarone has been used to treat gout for decades, but it was withdrawn from some markets in 2003 due to hepatotoxicity [126]. It is a highly promiscuous inhibitor that interacts with secretory transporters OAT1, OAT3, BCRP, MRP4 as well as reuptake transporters URAT1 and OAT4 to a clinically significant extent (Table 3). It inhibits URAT1, OAT3 and MRP4 at similar potencies (Table 4). The marked uricosuric effect of the drug clearly shows the dominant role of URAT1 in renal urate homeostasis.

#### 5.2.2. Probenecid

Interestingly, probenecid inhibits OAT1 and OAT3 more potently than URAT1 or OAT4 (Table 3 and Table 4). However, at the C_max,u_ of 46.9 µM it likely fully inhibits reabsorption of urate via URAT1 and OAT4.

#### 5.2.3. Sulfinpyrazone

Sulfinpyrazone is one of the oldest drugs used to treat hyperuricemia [127]. It most potently inhibits MRP4 but clinically significant inhibition of URAT1 is thought to be behind the uricosuric effect of the drug (Table 3 and Table 4).

#### 5.2.4. Losartan

Losartan is an angiotensin II receptor blocker (ARB) and thus not a *bona fide* uricosuric agent. However, it inhibits URAT1 about 100- to 780-fold more potently than the secretory urate transporters MRP4, OAT1 and OAT3 (Table 4) and displays a marked uricosuric effect clinically [128]. Other ARBs, including pratosartan and telmisartan, have also been shown to inhibit URAT1 along with both OAT4 and secretory OATs with varying potencies in vitro [101]. However, a clinical uricosuric effect of any ARB other than losartan has not been documented.

#### 5.2.5. Salicylic Acid

Salicylic acid has a biphasic effect as it increases SU levels at low doses but decreases SU levels at high doses [103]. A mechanistic investigation showed that salicylate at low concentrations stimulated URAT1-mediated urate uptake as it acted as a counterion. At higher doses, however, salicylate inhibited URAT1-mediated urate transport [104]. It was suggested that at low doses the trans-stimulatory effect was dominant over the cis-inhibitory effect on URAT1-mediated urate transport [104].

#### 5.2.6. Lesinurad

Lesinurad inhibited the apical reuptake transporters URAT1 and OAT4, as well as the basolateral transporters OAT1 and OAT3 involved in the secretory transport of urate, with similar IC50 values (Table 3 and Table 4). Using the C_max,u_ /IC_50_ ≥ 0.1 cut-off [129] both inhibitions were of clinical significance. However, lesinurad is an OAT1 and OAT3 substrate [94]; hence, intracellular concentrations are expected to be higher than plasma concentrations and, therefore, a greater degree of inhibition of URAT1 and OAT4 is likely. Perhaps this is reflected in the decision by PMDA to set a five-fold lower (C_max,u_ /IC_50_ ≥ 0.02) cut-off value for apical compared to basolateral transporters of PTC in their respective guidance document. Lesinurad also inhibits intestinal BCRP to a clinically significant extent (Table 3). Lesinurad was the first drug approved specifically for targeting URAT1, although, as described above, it inhibits a broad range of intestinal and renal secretory urate transporters as well. It also potently inhibits OAT4, which may complement its uricosuric action exerted through the blockade of URAT1.

#### 5.2.7. Dotinurad

Dotinurad was the second drug to be approved for targeting URAT1, and termed a selective urate reabsorption inhibitor for its ability to potently inhibit URAT1 without markedly affecting urate secretion transporters [69]. Indeed, dotinurad inhibited URAT1 110-fold and 35.5-fold more potently than OAT1 and OAT3 (Table 4), respectively [69]. Inhibition of URAT1 but not of OAT1 and OAT3 was of clinical significance (Table 3). Inhibition of BCRP in the gut was just above the cut-off (C_gut_/IC_50_ = 10.8). This, again, confirms that inhibition of renal urate reabsorption dominates in the antihyperuricemic effect of uricosuric drugs.

#### 5.2.8. Verinurad

Verinurad is a drug in clinical development also specific for URAT1. Verinurad inhibited URAT1 about 200-fold more potently than OAT1 or OAT4 (Table 4). Inhibition of URAT1 but not of OAT1 or OAT4 was of clinical significance (Table 3). Verinurad is also an OAT1 and OAT3 substrate [96]. Thus, inhibition of URAT1 is likely even greater than a C_max,u_/IC_50_ value of 0.37 would suggest.

#### 5.2.9. Tranilast

Tranilast was originally identified as an anti-allergic agent, but its additional benefits were later discovered in a variety of diseases [130]. More recently, it was shown to inhibit the transporters URAT1, OAT4, OAT10 and GLUT9, which mediate urate reabsorption (Table 3). In fact, tranilast has an even broader interaction profile as it also inhibits the secretory urate transporters OAT3 and NPT1 at about the same potency as renal reabsorptive transporters (Table 3 and Table 4). It fully inhibits OAT1 at 100 µM but no IC_50_ or K_i_ data are available for that interaction. Tranilast is highly protein-bound and at the calculated value of C_max,u_ = 0.57 µM the C_max,u_/IC_50_ ≥ 0.1 cut-off did not predict clinically relevant interactions for basolateral OAT3. The C_max,u_/IC_50_ ≥ 0.02 cut-off did predict clinically relevant interactions for both OAT3 and apical transporters NPT1, URAT1, OAT4, GLUT9. Based on formally calculated cut-offs, the clinical significance of these interactions was borderline. However, tranilast as a carboxylic acid may be an OAT1 and/or OAT3 substrate and thus may reach higher intracellular concentrations, leading to more significant inhibition of apical transporters, including reabsorptive ones.

#### 5.2.10. Arhalofenate

Arhalofenate inhibits multiple urate reuptake transporters, most pronouncedly OAT4 with a C_max,u_/IC_50_ value of 1.3. C_max,u_/IC_50_ values for inhibition of URAT1 and OAT10 (0.037 and 0.064, respectively) can only be considered clinically significant if the C_max,u_/IC_50_ ≥ 0.02 cut-off is used. No data are available in public databases on the effect of arhalofenate on secretory urate transporters.

#### 5.2.11. Fenofibrate

Fenofibrate is a lipid-lowering agent that is promptly hydrolyzed to its active metabolite fenofibric acid [131]. Fenofibrate is an inhibitor of BCRP, but this effect is unlikely to be clinically significant (Table 3). Fenofibric acid is a more potent inhibitor of OAT3 than of URAT1 (Table 3 and Table 4). If the C_max,u_/(IC_50_ or K_i_) > 0.02 criteria is used, both inhibitions are predicted to be clinically significant (Table 3).

All uricosuric agents with a reported BCRP IC_50_ or K_i_ (benzbromarone, lesinurad and dotinurad) displayed clinically significant inhibition of BCRP in the gut (Table 3). As this interaction acts oppositely to the inhibition of renal reabsorptive transporters yet does not cancel the clinically observed urate-lowering effect of these drugs, it can be speculated that intestinal secretion of urate by BCRP probably plays a minor role in the overall urate balance.

Due to their dominant role in determining SU levels, renal reabsorption transporters were chosen to evaluate whether the cut-offs recommended by regulatory guidelines, i.e., C_max,u_/(IC_50_ or K_i_) ≥ 0.1 or ≥0.02, are useful for the prediction of a clinically relevant antihyperuricemic effect based on in vitro transporter inhibition data (Table 3). Known uricosuric agents were considered positive controls, as they were assumed to decrease SU levels to a clinically relevant extent. The cut-off of C_max,u_/(IC_50_ or K_i_) ≥ 0.02 was a somewhat better indication of uricosuric efficacy than C_max,u_/(IC_50_ or K_i_) ≥ 0.1, as 10 of 10 uricosuric agents showed a clinically significant inhibition of URAT1 when the cut-off ≥ 0.02 was used, compared to 8 of 10 with the cut-off of ≥0.1 (Table 3). For OAT4, the hit rate was five of seven with the ≥0.02 cut-off and two of seven with the ≥0.1 cut-off. For OAT10 and GLUT9, only one of three drugs showed clinically significant inhibition and both were classified as positives according to the ≥0.02 cut-off (Table 3). Therefore, the more conservative C_max,u_/(IC_50_ or K_i_) ≥ 0.02 cut-off seems to provide greater accuracy.

### 5.3. Dual Inhibitors

Development of dual inhibitors (i.e., inhibitors of XO and URAT1) is an alternative to combination treatment with XO inhibitors and URAT1 inhibitors. Several inhibitors targeting both proteins are in development [76]. Transporter interaction information, however, is only known for URC-102 (epaminurad), which was described as a selective inhibitor of URAT1 as it inhibits URAT1 more potently than OAT1 or OAT2 [132].

## 6. Drug-Induced Hyperuricemia

Most drugs well known to cause hyperuricemia such as bumetanide [133], furosemide [134], chlorothiazide [135,136], hydrochlorothiazide [137], bendrofluazide [138] and torasemide [139] are diuretics. Bumetanide, furosemide and torasemide are loop diuretics, while chlorothiazide, hydrochlorothiazide and bendroflumethiazide are thiazide diuretics [140]. Pharmacokinetic properties of hyperuricemic drugs discussed in this review are listed in Table 2.

All diuretics inhibited at least one transporter participating in urate efflux to a clinically significant extent (Table 5); for bendroflumethiazide, inhibition data of murine Oat1 and Oat3 was available only. Chlorothiazide inhibited all secretory urate pathways tested (OAT1, OAT3, BCRP_kidney_, BCRP_gut_, NPT4). Data on URAT1 inhibition are only available for furosemide. As the C_max,u_ of furosemide is 0.697 µM (Table 2), the 71.6% inhibition at the concentration of 1 mM is unlikely to be clinically significant [18]. In addition, thiazide diuretics are thought to potentiate urate reabsorption as reduced plasma volume and accelerated sodium excretion activates the renin–angiotensin system (RAS) via slowing renal blood flow [141]. It has also been suggested that activation of the RAS and hyperosmolarity may lead to overexpression of URAT1 [141]. A similar scenario can be envisioned for loop diuretics, since they also activate the RAS [142,143]. Although the overexpression of URAT1 upon treatment with diuretics has not been documented in public databases, upregulation of the sodium–hydrogen exchanger NHE3 upon increased angiotensin II has been reported [144]. Increased NHE3 leads to acidification of the lumen and URAT1 activity is increased at acidic pH [5].

The hyperuricemic effect of low dose salicylate [104,125] and the pyrazinamide metabolite pyrazinoic acid (PZA) [104] is also centered around URAT1, as both compounds are thought to work through trans-stimulation of URAT1-mediated urate reabsorption. A similar mechanism was suggested for the M1 metabolite of favipiravir that was shown to increase URAT1-mediated urate reuptake [145]. This stimulatory effect and the inhibition of secretory urate transporters by favipiravir and M1 may override inhibition of URAT1 by favipiravir or M1. The clinical significance of these interactions is difficult to assess as IC_50_ values are not available.

Trans-stimulation of OAT4-mediated transport was suggested for torasemide and two of its metabolites [146], and trans-stimulation of OAT10 by cyclosporine A (CsA) has been described [59]. Therefore, trans-stimulation of reabsorptive transporters including URAT1, OAT4 and OAT10 is a mechanism that may need to be evaluated in preloading type experiments. For small hydrophilic compounds such as salicylic acid, PZA, torasemide and metabolites, and the favipiravir metabolite M1, an exchange mechanism has been proposed. CsA, however, is a large and more lipophilic compound, and thus an exchange mechanism seems unlikely.

In summary, all drugs known to cause hyperuricemia showed a clinically significant inhibition of at least one of the transporters participating in urate efflux (Table 5). The more conservative C_max,u_/(IC_50_ or K_i_) ≥ 0.02 provided only a slight edge over the C_max,u_/(IC_50_ or K_i_) ≥ 0.1 cut-off as it predicted clinically significant inhibition of OAT3 by furosemide, inhibition of BCRP by cyclosporine A and inhibition of NPT4 by chlorothiazide. No IC_50_ or K_i_ data were publicly available for inhibition of URAT1 by these compounds.

**Table 5:** Transporter interaction profile of hyperuricemic agents. For each drug/transporter pair, results from different studies are separated by semicolons. All values are in µM unless noted otherwise. N/I, no inhibition; TS, trans-stimulation; (H)/(L), high/low affinity transport. The rules for highlighting in **bold** or **bold and underlined** are the same as in Table 3. Values in red were obtained using urate as a probe substrate. Abbreviations: Secr., secretion; Reabs., reabsorption.

Transporter	BCRP (Gut)	OAT1	OAT3	BCRP (Kidney)	MRP4	NPT4	URAT1	OAT4	OAT10	GLUT9	
Role	Secr.	Secr.	Secr.	Secr.	Secr.	Secr.	Reabs.	Reabs.	Reabs.	Reabs.	
Drug/Compound											References
**Bumetanide**	IC50 = ~100–1000	IC50 = **1.9** (rOat1);7.60	IC50 = **0.75**	IC50 = ~100–1000	N/I; substrate; IC50 = ~ 10 -100	IC50 = 223.5		IC50 = 348			[51,71,72,147,148,149]
**Furosemide**	IC50 = 170	IC50 = **5.05**; **18**	IC50 = 51.1; **7.31**	IC50 = 170	IC50 = **1.29**	IC50 = 73.5	71.6% inhibition at 1 mM	IC50 = 44.5			[18,51,67,70,71,148]
**Torasemide**		Ki = 55.2	Ki = 89.9; TS of E3S transport				N/I	Ki = 47.0; TS of urate transport			[146]
**Chlorothiazide**	IC50 = **212.3**	IC50 = **3.78**	IC50 = **65.3**	IC50 = **212.3**	IC50 = **0.24****nM (H)/****10.4****(L)**	IC50 = **739.6**		IC50 = 2632			[51,71,148,150]
**Hydrochlorothiazide**	N/I	IC50 = 126	IC50 = 213	N/I	IC50 = **1.9****(H)**/220 (L)			TS of urate uptake			[58,71,151]
**Bendroflumethiazide**		IC50 = 8 (mOat1)	IC50 = 21 (mOat3)								[152]
**Salicylate (low dose)**		IC50 = **1573.4**; Ki = **341**			IC50 = **2.1****(H)****/1547 (L)**		TS of urate uptake				[34,71,75,103,104,125]
**Pyrazinoate**		IC50 = 582.6					N/I; TS of urate uptake				[18,34,104]
**Cyclosporine A**	IC50 = 4.6	N/I	N/I	IC50 = **4.6**	N/I				TS of urate uptake		[59,73,153]
**Favipiravir**		30.9% inhibition at 800 µM	50.0% inhibition at 800 µM				65.7% inhibition at 800 µM				[145]
**Favipiravir M1**		45.4% inhibition at 300 µM	57.7% inhibition at 300 µM				31.0% inhibition at 300 µM; stimulation of urate uptake				[145]

## 7. Conclusions

The inhibition profiles of renal transporters as well as of intestinal BCRP have been instrumental in understanding the effect of drugs on serum urate levels. Due to the relatively low protein binding of urate, the majority of urate undergoes glomerular filtration and reabsorption. It appears that URAT1 plays a key role in urate reabsorption and in the maintenance of serum urate levels. A recent study demonstrated hypouricemia in a patient homozygous for the Trp258* (rs121907892) mutation in URAT1 despite being heterozygous for a variant of BCRP (ABCG2 Gln141Lys (rs2231142)) as well as homozygous for a mutation in NPT1 (SLC17A1 Thr269Ile (rs1165196)), both known to cause hyperuricemia [154]. In addition, four of six uricosuric agents (Table 3) but only two of five hyperuricemic drugs (Table 5) with available BCRP inhibition data showed clinically significant inhibition of gut BCRP, suggesting a less prominent role for gut-localized BCRP than results with *Abcg2* knockout mice would suggest [3]. These observations confirm the dominance of urate reuptake via URAT1 over urate secretion in the general population, albeit intestinal BCRP may gain in relative importance in chronic kidney disease patients, where the strongest association between ABCG2 SNPs and increased serum urate was observed [155]. Most uricosuric drugs inhibit URAT1 more potently than the transporters implicated in secretory transport of urate such as OAT1, OAT3, BCRP, MRP4, NPT1, or NPT4. Diuretics linked to hyperuricemia have been shown to inhibit secretory functions but have not been tested for inhibition of URAT1. Two hyperuricemic drugs, low dose salicylic acid and pyrazinamide, as well as the M1 metabolite of favipiravir, have been shown to facilitate URAT1-mediated urate reabsorption, once again supporting a key role for URAT1. In sum, urate homeostasis hinges on a delicate balance of secretory and reabsorptive transport, and inhibition profiling of drugs across a relevant panel of transporters may shed light on their potential to interfere with urate disposition. Hyperuricemia is generally considered a more serious outcome than hypouricemia, but the latter may become more prevalent as ever more potent URAT1 inhibitors are being discovered. Therefore, in the safety assessment of drug candidates with respect to urate transport, attention should be paid both to the inhibition of renal secretory urate transporters and the inhibition or trans-stimulation of renal reabsorptive transporters. These interactions could be screened in vitro early in development, and in case the results suggest a major imbalance, e.g., a pronounced bias towards the inhibition of secretion and/or stimulation of reabsorption, alternative drug candidates with more balanced interaction profiles might be prioritized.

## Figures and Tables

**Table 1:** Transporters involved in urate disposition. Abbreviations: 5-/6-CF, 5-/6-carboxyfluorescein; DHEAS, dehydroepiandrosterone sulfate; E3S, estrone 3-sulfate; MTX, methotrexate; PAH, *p*-aminohippuric acid; PhA, pheophorbide A; PTC, proximal tubule cells; SU, serum urate.

Transporter Common Name/*Gene Symbol*	Known Genetic Association with SU	Localization in the PTC	Cellular Direction of Transport	Function in Urate Disposition	Probe Substrates Other than Urate Used in In Vitro Interaction Assays
OAT1/***SLC22A6***	Y	basolateral	uptake	secretion	PAH, 6-CF, chlorothiazide, MTX
OAT2/***SLC22A7***	Y	basolateral	uptake	secretion	-
OAT3/***SLC22A8***	N	basolateral	uptake	secretion	PAH, E3S, 5-CF, MTX
BCRP/***ABCG2***	Y	apical	efflux	secretion	genistein, PhA, E3S, MTX, rosuvastatin
MRP4/***ABCC4***	Y	apical	efflux	secretion	DHEAS, MTX
NPT1/***SLC17A1***	Y	apical	efflux	secretion	-
NPT4/***SLC17A3***	Y	apical	efflux	secretion	-
URAT1/***SLC22A12***	Y	apical	uptake	reabsorption	-
OAT4/***SLC22A11***	Y	apical	uptake	reabsorption	E3S
OAT10/***SLC22A13***	N	apical	uptake	reabsorption	-
GLUT9/***SLC2A9***	Y	basolateral	efflux	reabsorption	-

## References

[1] Wu X.W., Muzny D.M., Lee C.C., Caskey C.T. (1992). Two independent mutational events in the loss of urate oxidase during hominoid evolution. J. Mol. Evol..

[2] Sattui S.E., Gaffo A.L. (2016). Treatment of hyperuricemia in gout: Current therapeutic options, latest developments and clinical implications. Ther. Adv. Musculoskelet. Dis..

[3] Hosomi A., Nakanishi T., Fujita T., Tamai I. (2012). Extra-renal elimination of uric acid via intestinal efflux transporter BCRP/ABCG2. PLoS ONE.

[4] Bertolini J., Masarei J.R. (1979). The binding of urate by plasma proteins. Aust. J. Exp. Biol. Med. Sci..

[5] Hyndman D., Liu S., Miner J.N. (2016). Urate Handling in the Human Body. Curr. Rheumatol. Rep..

[6] Becker M.A., Schumacher H.R., Wortmann R.L., MacDonald P.A., Eustace D., Palo W.A., Streit J., Joseph-Ridge N. (2005). Febuxostat compared with allopurinol in patients with hyperuricemia and gout. N. Engl. J. Med..

[7] Zhang W., Doherty M., Bardin T., Pascual E., Barskova V., Conaghan P., Gerster J., Jacobs J., Leeb B., Liote F. (2006). EULAR evidence based recommendations for gout. Part II: Management. Report of a task force of the EULAR Standing Committee for International Clinical Studies Including Therapeutics (ESCISIT). Ann. Rheum. Dis..

[8] Perez-Ruiz F., Liote F. (2007). Lowering serum uric acid levels: What is the optimal target for improving clinical outcomes in gout?. Arthritis Rheum..

[9] Nakayama A., Nakatochi M., Kawamura Y., Yamamoto K., Nakaoka H., Shimizu S., Higashino T., Koyama T., Hishida A., Kuriki K. (2020). Subtype-specific gout susceptibility loci and enrichment of selection pressure on ABCG2 and ALDH2 identified by subtype genome-wide meta-analyses of clinically defined gout patients. Ann. Rheum. Dis..

[10] Hong F., Zheng A., Xu P., Wang J., Xue T., Dai S., Pan S., Guo Y., Xie X., Li L. (2020). High-Protein Diet Induces Hyperuricemia in a New Animal Model for Studying Human Gout. Int. J. Mol. Sci..

[11] Richette P., Bardin T. (2010). Gout. Lancet.

[12] Zhu Y., Pandya B.J., Choi H.K. (2011). Prevalence of gout and hyperuricemia in the US general population: The National Health and Nutrition Examination Survey 2007–2008. Arthritis Rheum..

[13] Liu F., Du G.L., Song N., Ma Y.T., Li X.M., Gao X.M., Yang Y.N. (2020). Hyperuricemia and its association with adiposity and dyslipidemia in Northwest China: Results from cardiovascular risk survey in Xinjiang (CRS 2008–2012). Lipids Health Dis..

[14] Konta T., Ichikawa K., Kawasaki R., Fujimoto S., Iseki K., Moriyama T., Yamagata K., Tsuruya K., Narita I., Kondo M. (2020). Association between serum uric acid levels and mortality: A nationwide community-based cohort study. Sci. Rep..

[15] George C., Minter D.A. (2020). Hyperuricemia.

[16] Pineda C., Soto-Fajardo C., Mendoza J., Gutierrez J., Sandoval H. (2020). Hypouricemia: What the practicing rheumatologist should know about this condition. Clin. Rheumatol..

[17] Nakayama A., Matsuo H., Ohtahara A., Ogino K., Hakoda M., Hamada T., Hosoyamada M., Yamaguchi S., Hisatome I., Ichida K. (2019). Clinical practice guideline for renal hypouricemia (1st edition). Hum. Cell.

[18] Enomoto A., Kimura H., Chairoungdua A., Shigeta Y., Jutabha P., Cha S.H., Hosoyamada M., Takeda M., Sekine T., Igarashi T. (2002). Molecular identification of a renal urate anion exchanger that regulates blood urate levels. Nature.

[19] Dinour D., Gray N.K., Campbell S., Shu X., Sawyer L., Richardson W., Rechavi G., Amariglio N., Ganon L., Sela B.A. (2010). Homozygous SLC2A9 mutations cause severe renal hypouricemia. J. Am. Soc. Nephrol..

[20] Li Z., Ding H., Chen C., Chen Y., Wang D.W., Lv Y. (2013). Novel URAT1 mutations caused acute renal failure after exercise in two Chinese families with renal hypouricemia. Gene.

[21] Jeannin G., Chiarelli N., Gaggiotti M., Ritelli M., Maiorca P., Quinzani S., Verzeletti F., Possenti S., Colombi M., Cancarini G. (2014). Recurrent exercise-induced acute renal failure in a young Pakistani man with severe renal hypouricemia and SLC2A9 compound heterozygosity. BMC Med. Genet..

[22] Wang Z., Cui T., Ci X., Zhao F., Sun Y., Li Y., Liu R., Wu W., Yi X., Liu C. (2019). The effect of polymorphism of uric acid transporters on uric acid transport. J. Nephrol..

[23] So A., Thorens B. (2010). Uric acid transport and disease. J. Clin. Investig..

[24] Fatima T., McKinney C., Major T.J., Stamp L.K., Dalbeth N., Iverson C., Merriman T.R., Miner J.N. (2018). The relationship between ferritin and urate levels and risk of gout. Arthritis Res. Ther..

[25] So A.K., Martinon F. (2017). Inflammation in gout: Mechanisms and therapeutic targets. Nat. Rev. Rheumatol..

[26] Bobulescu I.A., Moe O.W. (2012). Renal transport of uric acid: Evolving concepts and uncertainties. Adv. Chronic Kidney Dis..

[27] Ponticelli C., Podesta M.A., Moroni G. (2020). Hyperuricemia as a trigger of immune response in hypertension and chronic kidney disease. Kidney Int..

[28] Lanaspa M.A., Andres-Hernando A., Kuwabara M. (2020). Uric acid and hypertension. Hypertens. Res. Off. J. Jpn. Soc. Hypertens..

[29] Feig D.I., Soletsky B., Johnson R.J. (2008). Effect of allopurinol on blood pressure of adolescents with newly diagnosed essential hypertension: A randomized trial. JAMA.

[30] Stewart D.J., Langlois V., Noone D. (2019). Hyperuricemia and Hypertension: Links and Risks. Integr. Blood Press. Control.

[31] Ichida K., Matsuo H., Takada T., Nakayama A., Murakami K., Shimizu T., Yamanashi Y., Kasuga H., Nakashima H., Nakamura T. (2012). Decreased extra-renal urate excretion is a common cause of hyperuricemia. Nat. Commun..

[32] Xu X., Li C., Zhou P., Jiang T. (2016). Uric acid transporters hiding in the intestine. Pharm. Biol..

[33] Otani N., Ouchi M., Hayashi K., Jutabha P., Anzai N. (2017). Roles of organic anion transporters (OATs) in renal proximal tubules and their localization. Anat. Sci. Int..

[34] Ichida K., Hosoyamada M., Kimura H., Takeda M., Utsunomiya Y., Hosoya T., Endou H. (2003). Urate transport via human PAH transporter hOAT1 and its gene structure. Kidney Int..

[35] Bakhiya A., Bahn A., Burckhardt G., Wolff N. (2003). Human organic anion transporter 3 (hOAT3) can operate as an exchanger and mediate secretory urate flux. Cell. Physiol. Biochem. Int. J. Exp. Cell. Physiol. Biochem. Pharmacol..

[36] Eraly S.A., Vallon V., Rieg T., Gangoiti J.A., Wikoff W.R., Siuzdak G., Barshop B.A., Nigam S.K. (2008). Multiple organic anion transporters contribute to net renal excretion of uric acid. Physiol. Genom..

[37] Kanai M., Akiyama M., Takahashi A., Matoba N., Momozawa Y., Ikeda M., Iwata N., Ikegawa S., Hirata M., Matsuda K. (2018). Genetic analysis of quantitative traits in the Japanese population links cell types to complex human diseases. Nat. Genet..

[38] Zou L., Stecula A., Gupta A., Prasad B., Chien H.C., Yee S.W., Wang L., Unadkat J.D., Stahl S.H., Fenner K.S. (2018). Molecular Mechanisms for Species Differences in Organic Anion Transporter 1, OAT1: Implications for Renal Drug Toxicity. Mol. Pharmacol..

[39] Henjakovic M., Hagos Y., Krick W., Burckhardt G., Burckhardt B.C. (2015). Human organic anion transporter 2 is distinct from organic anion transporters 1 and 3 with respect to transport function. Am. J. Physiol. Ren. Physiol..

[40] Oswald S., Muller J., Neugebauer U., Schroter R., Herrmann E., Pavenstadt H., Ciarimboli G. (2019). Protein Abundance of Clinically Relevant Drug Transporters in The Human Kidneys. Int. J. Mol. Sci..

[41] Nagy I., Toth B., Gaborik Z., Erdo F., Krajcsi P. (2016). Membrane Transporters in Physiological Barriers of Pharmacological Importance. Curr. Pharm. Des..

[42] Ray K.K., Bakris G.L., Banach M., Catapano A., Duell P.B., Mancini G.B.J., Bloedon L., Feng A., Gotto A.M. (2020). Effect of bempedoic acid on uric acid and gout in 3621 patients with hypercholesterolemia: Pooled analyses from phase 3 trials. Eur. Heart J..

[43] Matsuo H., Takada T., Ichida K., Nakamura T., Nakayama A., Ikebuchi Y., Ito K., Kusanagi Y., Chiba T., Tadokoro S. (2009). Common defects of ABCG2, a high-capacity urate exporter, cause gout: A function-based genetic analysis in a Japanese population. Sci. Transl. Med..

[44] Wada S., Matsunaga N., Tamai I. (2020). Mathematical modeling analysis of hepatic uric acid disposition using human sandwich-cultured hepatocytes. Drug Metab. Pharmacokinet..

[45] Ristic B., Sikder M.O.F., Bhutia Y.D., Ganapathy V. (2020). Pharmacologic inducers of the uric acid exporter ABCG2 as potential drugs for treatment of gouty arthritis. Asian J. Pharm. Sci..

[46] van Aubel R.A., Smeets P.H., Peters J.G., Bindels R.J., Russel F.G. (2002). The MRP4/ABCC4 gene encodes a novel apical organic anion transporter in human kidney proximal tubules: Putative efflux pump for urinary cAMP and cGMP. J. Am. Soc. Nephrol..

[47] Van Aubel R.A., Smeets P.H., van den Heuvel J.J., Russel F.G. (2005). Human organic anion transporter MRP4 (ABCC4) is an efflux pump for the purine end metabolite urate with multiple allosteric substrate binding sites. Am. J. Physiol. Ren. Physiol..

[48] Tanner C., Boocock J., Stahl E.A., Dobbyn A., Mandal A.K., Cadzow M., Phipps-Green A.J., Topless R.K., Hindmarsh J.H., Stamp L.K. (2017). Population-Specific Resequencing Associates the ATP-Binding Cassette Subfamily C Member 4 Gene With Gout in New Zealand Maori and Pacific Men. Arthritis Rheumatol..

[49] Reimer R.J., Edwards R.H. (2004). Organic anion transport is the primary function of the SLC17/type I phosphate transporter family. Pflug. Arch. Eur. J. Physiol..

[50] Iharada M., Miyaji T., Fujimoto T., Hiasa M., Anzai N., Omote H., Moriyama Y. (2010). Type 1 sodium-dependent phosphate transporter (SLC17A1 Protein) is a Cl(-)-dependent urate exporter. J. Biol. Chem..

[51] Jutabha P., Anzai N., Wempe M.F., Wakui S., Endou H., Sakurai H. (2011). Apical voltage-driven urate efflux transporter NPT4 in renal proximal tubule. Nucleosidesnucleotides Nucleic Acids.

[52] Sakiyama M., Matsuo H., Nagamori S., Ling W., Kawamura Y., Nakayama A., Higashino T., Chiba T., Ichida K., Kanai Y. (2016). Expression of a human NPT1/SLC17A1 missense variant which increases urate export. Nucleosides Nucleotides Nucleic Acids.

[53] Chiba T., Matsuo H., Kawamura Y., Nagamori S., Nishiyama T., Wei L., Nakayama A., Nakamura T., Sakiyama M., Takada T. (2015). NPT1/SLC17A1 is a renal urate exporter in humans and its common gain-of-function variant decreases the risk of renal underexcretion gout. Arthritis Rheumatol..

[54] Ichida K., Hosoyamada M., Hisatome I., Enomoto A., Hikita M., Endou H., Hosoya T. (2004). Clinical and molecular analysis of patients with renal hypouricemia in Japan-influence of URAT1 gene on urinary urate excretion. J. Am. Soc. Nephrol..

[55] Komoda F., Sekine T., Inatomi J., Enomoto A., Endou H., Ota T., Matsuyama T., Ogata T., Ikeda M., Awazu M. (2004). The W258X mutation in SLC22A12 is the predominant cause of Japanese renal hypouricemia. Pediatric Nephrol..

[56] Hosoyamada M., Takiue Y., Morisaki H., Cheng J., Ikawa M., Okabe M., Morisaki T., Ichida K., Hosoya T., Shibasaki T. (2010). Establishment and analysis of SLC22A12 (URAT1) knockout mouse. Nucleosidesnucleotides Nucleic Acids.

[57] Nigam S.K. (2018). The SLC22 Transporter Family: A Paradigm for the Impact of Drug Transporters on Metabolic Pathways, Signaling, and Disease. Annu. Rev. Pharmacol. Toxicol..

[58] Hagos Y., Stein D., Ugele B., Burckhardt G., Bahn A. (2007). Human renal organic anion transporter 4 operates as an asymmetric urate transporter. J. Am. Soc. Nephrol..

[59] Bahn A., Hagos Y., Reuter S., Balen D., Brzica H., Krick W., Burckhardt B.C., Sabolic I., Burckhardt G. (2008). Identification of a new urate and high affinity nicotinate transporter, hOAT10 (SLC22A13). J. Biol. Chem..

[60] Sakiyama M., Matsuo H., Shimizu S., Nakashima H., Nakayama A., Chiba T., Naito M., Takada T., Suzuki H., Hamajima N. (2014). A common variant of organic anion transporter 4 (OAT4/SLC22A11) gene is associated with renal underexcretion type gout. Drug Metab. Pharmacokinet..

[61] Higashino T., Morimoto K., Nakaoka H., Toyoda Y., Kawamura Y., Shimizu S., Nakamura T., Hosomichi K., Nakayama A., Ooyama K. (2020). Dysfunctional missense variant of OAT10/SLC22A13 decreases gout risk and serum uric acid levels. Ann. Rheum. Dis..

[62] Caulfield M.J., Munroe P.B., O’Neill D., Witkowska K., Charchar F.J., Doblado M., Evans S., Eyheramendy S., Onipinla A., Howard P. (2008). SLC2A9 is a high-capacity urate transporter in humans. PLoS Med..

[63] Wright A.F., Rudan I., Hastie N.D., Campbell H. (2010). A ‘complexity’ of urate transporters. Kidney Int..

[64] Anzai N., Ichida K., Jutabha P., Kimura T., Babu E., Jin C.J., Srivastava S., Kitamura K., Hisatome I., Endou H. (2008). Plasma urate level is directly regulated by a voltage-driven urate efflux transporter URATv1 (SLC2A9) in humans. J. Biol. Chem..

[65] Major T.J., Dalbeth N., Stahl E.A., Merriman T.R. (2018). An update on the genetics of hyperuricaemia and gout. Nat. Rev. Rheumatol..

[66] Preitner F., Bonny O., Laverriere A., Rotman S., Firsov D., Da Costa A., Metref S., Thorens B. (2009). Glut9 is a major regulator of urate homeostasis and its genetic inactivation induces hyperuricosuria and urate nephropathy. Proc. Natl. Acad. Sci. USA.

[67] Ebner T., Ishiguro N., Taub M.E. (2015). The Use of Transporter Probe Drug Cocktails for the Assessment of Transporter-Based Drug-Drug Interactions in a Clinical Setting-Proposal of a Four Component Transporter Cocktail. J. Pharm. Sci..

[68] Mandal A.K., Mercado A., Foster A., Zandi-Nejad K., Mount D.B. (2017). Uricosuric targets of tranilast. Pharmacol. Res. Perspect..

[69] Taniguchi T., Ashizawa N., Matsumoto K., Saito R., Motoki K., Sakai M., Chikamatsu N., Hagihara C., Hashiba M., Iwanaga T. (2019). Pharmacological Evaluation of Dotinurad, a Selective Urate Reabsorption Inhibitor. J. Pharmacol. Exp. Ther..

[70] Juhasz V., Beery E., Nagy Z., Bui A., Molnar E., Zolnerciks J.K., Magnan R., Jani M., Krajcsi P. (2013). Chlorothiazide is a substrate for the human uptake transporters OAT1 and OAT3. J. Pharm. Sci..

[71] El-Sheikh A.A., van den Heuvel J.J., Koenderink J.B., Russel F.G. (2008). Effect of hypouricaemic and hyperuricaemic drugs on the renal urate efflux transporter, multidrug resistance protein 4. Br. J. Pharmacol..

[72] Hasegawa M., Kusuhara H., Adachi M., Schuetz J.D., Takeuchi K., Sugiyama Y. (2007). Multidrug resistance-associated protein 4 is involved in the urinary excretion of hydrochlorothiazide and furosemide. J. Am. Soc. Nephrol..

[73] Poirier A., Portmann R., Cascais A.C., Bader U., Walter I., Ullah M., Funk C. (2014). The need for human breast cancer resistance protein substrate and inhibition evaluation in drug discovery and development: Why, when, and how?. Drug Metab. Dispos. Biol. Fate Chem..

[74] Weiss J., Sauer A., Divac N., Herzog M., Schwedhelm E., Boger R.H., Haefeli W.E., Benndorf R.A. (2010). Interaction of angiotensin receptor type 1 blockers with ATP-binding cassette transporters. Biopharm. Drug Dispos..

[75] Apiwattanakul N., Sekine T., Chairoungdua A., Kanai Y., Nakajima N., Sophasan S., Endou H. (1999). Transport properties of nonsteroidal anti-inflammatory drugs by organic anion transporter 1 expressed in Xenopus laevis oocytes. Mol. Pharmacol..

[76] Otani N., Ouchi M., Kudo H., Tsuruoka S., Hisatome I., Anzai N. (2020). Recent approaches to gout drug discovery: An update. Expert Opin. Drug Discov..

[77] Diaz-Torne C., Perez-Herrero N., Perez-Ruiz F. (2015). New medications in development for the treatment of hyperuricemia of gout. Curr. Opin. Rheumatol..

[78] Yu Y., Zhang N., Dong X., Fan N., Wang L., Xu Y., Chen H., Duan W. (2020). Uricase-deficient rat is generated with CRISPR/Cas9 technique. PeerJ.

[79] Cai L., Li Q., Deng Y., Liu X., Du W., Jiang X. (2020). Construction and expression of recombinant uricaseexpressing genetically engineered bacteria and its application in rat model of hyperuricemia. Int. J. Mol. Med..

[80] Mahmoud H.H., Leverger G., Patte C., Harvey E., Lascombes F. (1998). Advances in the management of malignancy-associated hyperuricaemia. Br. J. Cancer.

[81] Pui C.H. (2001). Urate oxidase in the prophylaxis or treatment of hyperuricemia: The United States experience. Semin. Hematol..

[82] Bayol A., Capdevielle J., Malazzi P., Buzy A., Claude Bonnet M., Colloc’h N., Mornon J.P., Loyaux D., Ferrara P. (2002). Modification of a reactive cysteine explains differences between rasburicase and Uricozyme, a natural Aspergillus flavus uricase. Biotechnol. Appl. Biochem..

[83] Allen K.C., Champlain A.H., Cotliar J.A., Belknap S.M., West D.P., Mehta J., Trifilio S.M. (2015). Risk of anaphylaxis with repeated courses of rasburicase: A Research on Adverse Drug Events and Reports (RADAR) project. Drug Saf..

[84] Cunha R.N., Aguiar R., Farinha F. (2018). Impact of pegloticase on patient outcomes in refractory gout: Current perspectives. Open Access Rheumatol. Res. Rev..

[85] Sun S.S., Zhang D.H., Shi Y., Lin C.J., Lin J.Y. (2020). Efficacy and safety of urate-lowering treatments in patients with hyperuricemia: A comprehensive network meta-analysis of randomized controlled trials. J. Clin. Pharm. Ther..

[86] Carr A. (2016). Pharmacologic Treatment of Gout Now and in the Future. Pharmanote.

[87] Becker M.A., Hollister A.S., Terkeltaub R., Waugh A., Flynt A., Fitz-Patrick D., Sheridan W. (2013). FRI0367BCX4208 added to allopurinol increases response rates in patients with GOUT who fail to reach goal range serum uric acid on allopurinol alone: A randomized, double-blind, placebo-controlled trial. Ann. Rheum. Dis..

[88] Hollister A.S., Dobo S., Maetzel A., Becker M.A., Terkeltaub R., Fitz-Patrick D., Smith V., Sheridan W. (2013). FRI0380 Long-term safety of BCX4208 added to allopurinol in the chronic management of GOUT: Results of a phase 2 24-week blinded safety extension and vaccine challenge study. Ann. Rheum. Dis..

[89] Dong Y., Zhao T., Ai W., Zalloum W.A., Kang D., Wu T., Liu X., Zhan P. (2019). Novel urate transporter 1 (URAT1) inhibitors: A review of recent patent literature (2016–2019). Expert Opin. Ther. Pat..

[90] Benn C.L., Dua P., Gurrell R., Loudon P., Pike A., Storer R.I., Vangjeli C. (2018). Physiology of Hyperuricemia and Urate-Lowering Treatments. Front. Med..

[91] Uchida S., Shimada K., Misaka S., Imai H., Katoh Y., Inui N., Takeuchi K., Ishizaki T., Yamada S., Ohashi K. (2010). Benzbromarone pharmacokinetics and pharmacodynamics in different cytochrome P450 2C9 genotypes. Drug Metab. Pharmacokinet..

[92] Selen A., Amidon G.L., Welling P.G. (1982). Pharmacokinetics of probenecid following oral doses to human volunteers. J. Pharm. Sci..

[93] Pea F. (2005). Pharmacology of drugs for hyperuricemia. Mechanisms, kinetics and interactions. Contrib. Nephrol..

[94] Miner J.N., Tan P.K., Hyndman D., Liu S., Iverson C., Nanavati P., Hagerty D.T., Manhard K., Shen Z., Girardet J.L. (2016). Lesinurad, a novel, oral compound for gout, acts to decrease serum uric acid through inhibition of urate transporters in the kidney. Arthritis Res. Ther..

[95] Shen Z., Yeh L.T., Wallach K., Zhu N., Kerr B., Gillen M. (2016). In Vitro and In Vivo Interaction Studies Between Lesinurad, a Selective Urate Reabsorption Inhibitor, and Major Liver or Kidney Transporters. Clin. Drug Investig..

[96] Lee C.A., Yang C., Shah V., Shen Z., Wilson D.M., Ostertag T.M., Girardet J.L., Hall J., Gillen M. (2018). Metabolism and Disposition of Verinurad, a Uric Acid Reabsorption Inhibitor, in Humans. Drug Metab. Dispos. Biol. Fate Chem..

[97] Steinberg A.S., Vince B.D., Choi Y.J., Martin R.L., McWherter C.A., Boudes P.F. (2017). The Pharmacodynamics, Pharmacokinetics, and Safety of Arhalofenate in Combination with Febuxostat When Treating Hyperuricemia Associated with Gout. J. Rheumatol..

[98] Elsby R., Martin P., Surry D., Sharma P., Fenner K. (2016). Solitary Inhibition of the Breast Cancer Resistance Protein Efflux Transporter Results in a Clinically Significant Drug-Drug Interaction with Rosuvastatin by Causing up to a 2-Fold Increase in Statin Exposure. Drug Metab. Dispos. Biol. Fate Chem..

[99] Yamazaki M., Li B., Louie S.W., Pudvah N.T., Stocco R., Wong W., Abramovitz M., Demartis A., Laufer R., Hochman J.H. (2005). Effects of fibrates on human organic anion-transporting polypeptide 1B1-, multidrug resistance protein 2- and P-glycoprotein-mediated transport. Xenobiotica.

[100] Charng M.-J., Ding P.Y.-A., Chuang M.-H., Lo C.-Y., Chiang P.-S., Pao L.-H. (2002). Pharmacokinetic properties of tranilast in Chinese people. J. Food Drug Anal..

[101] Sato M., Iwanaga T., Mamada H., Ogihara T., Yabuuchi H., Maeda T., Tamai I. (2008). Involvement of uric acid transporters in alteration of serum uric acid level by angiotensin II receptor blockers. Pharm. Res..

[102] Vaidyanathan J., Yoshida K., Arya V., Zhang L. (2016). Comparing Various In Vitro Prediction Criteria to Assess the Potential of a New Molecular Entity to Inhibit Organic Anion Transporting Polypeptide 1B1. J. Clin. Pharmacol..

[103] Yu T.F., Gutman A.B. (1959). Study of the paradoxical effects of salicylate in low, intermediate and high dosage on the renal mechanisms for excretion of urate in man. J. Clin. Investig..

[104] Nakanishi T., Ohya K., Shimada S., Anzai N., Tamai I. (2013). Functional cooperation of URAT1 (SLC22A12) and URATv1 (SLC2A9) in renal reabsorption of urate. Nephrol. Dial. Transplant. Off. Publ. Eur. Dial. Transpl. Assoc. Eur. Ren. Assoc..

[105] Ahn S.O., Ohtomo S., Kiyokawa J., Nakagawa T., Yamane M., Lee K.J., Kim K.H., Kim B.H., Tanaka J., Kawabe Y. (2016). Stronger Uricosuric Effects of the Novel Selective URAT1 Inhibitor UR-1102 Lowered Plasma Urate in Tufted Capuchin Monkeys to a Greater Extent than Benzbromarone. J. Pharmacol. Exp. Ther..

[106] Holazo A.A., Colburn W.A., Gustafson J.H., Young R.L., Parsonnet M. (1984). Pharmacokinetics of bumetanide following intravenous, intramuscular, and oral administrations to normal subjects. J. Pharm. Sci..

[107] Shim H.J., Lee M.G., Lee M.H. (1991). Factors influencing the protein binding of bumetanide using an equilibrium dialysis technique. J. Clin. Pharm. Ther..

[108] FDA (2012). LASIX^®^. https://www.accessdata.fda.gov/drugsatfda_docs/label/2012/016273s066lbl.pdf.

[109] FDA (2010). Demadex. https://www.accessdata.fda.gov/drugsatfda_docs/label/2010/020136s023lbl.pdf.

[110] Knauf H., Mutschler E. (1998). Clinical pharmacokinetics and pharmacodynamics of torasemide. Clin. Pharmacokinet..

[111] Patel R.B., Patel U.R., Rogge M.C., Shah V.P., Prasad V.K., Selen A., Welling P.G. (1984). Bioavailability of hydrochlorothiazide from tablets and suspensions. J. Pharm. Sci..

[112] Welling P.G. (1986). Pharmacokinetics of the thiazide diuretics. Biopharm. Drug Dispos..

[113] Beermann B., Groschinsky-Grind M., Lindstrom B. (1977). Pharmacokinetics of bendroflumethiazide. Clin. Pharmacol. Ther..

[114] Harmon R.C., Enna S.J., Bylund D.B. (2007). Pyrazinamide. xPharm: The Comprehensive Pharmacology Reference.

[115] FDA (2009). NEORAL^®^. https://www.accessdata.fda.gov/drugsatfda_docs/label/2009/050715s027,050716s028lbl.pdf.

[116] Nguyen T.H., Guedj J., Anglaret X., Laouenan C., Madelain V., Taburet A.M., Baize S., Sissoko D., Pastorino B., Rodallec A. (2017). Favipiravir pharmacokinetics in Ebola-Infected patients of the JIKI trial reveals concentrations lower than targeted. PLoS Negl. Trop. Dis..

[117] Evaluation and Licensing Division, Pharmaceutical and Food Safety Bureau, Ministry of Health, Labour and Welfare (2014). Report on the Deliberation Results. https://www.pmda.go.jp/files/000210319.pdf.

[118] Wu T., Chen J., Dong S., Li H., Cao Y., Tian Y., Fu W., Zhou P., Xi B., Pang J. (2017). Identification and characterization of a potent and selective inhibitor of human urate transporter 1. Pharmacol. Rep..

[119] Lavan B.E., McWherter C., Choi Y.-J. (2013). FRI0403 Arhalofenate, a novel uricosuric agent, is an inhibitor of human URIC acid transporters. Ann. Rheum. Dis..

[120] Tan P.K., Liu S., Gunic E., Miner J.N. (2017). Discovery and characterization of verinurad, a potent and specific inhibitor of URAT1 for the treatment of hyperuricemia and gout. Sci. Rep..

[121] Chu X.Y., Bleasby K., Yabut J., Cai X., Chan G.H., Hafey M.J., Xu S., Bergman A.J., Braun M.P., Dean D.C. (2007). Transport of the dipeptidyl peptidase-4 inhibitor sitagliptin by human organic anion transporter 3, organic anion transporting polypeptide 4C1, and multidrug resistance P-glycoprotein. J. Pharmacol. Exp. Ther..

[122] Uetake D., Ohno I., Ichida K., Yamaguchi Y., Saikawa H., Endou H., Hosoya T. (2010). Effect of fenofibrate on uric acid metabolism and urate transporter 1. Intern. Med..

[123] Bibert S., Hess S.K., Firsov D., Thorens B., Geering K., Horisberger J.D., Bonny O. (2009). Mouse GLUT9: Evidences for a urate uniporter. Am. J. Physiol. Ren. Physiol..

[124] Shin H.J., Takeda M., Enomoto A., Fujimura M., Miyazaki H., Anzai N., Endou H. (2011). Interactions of urate transporter URAT1 in human kidney with uricosuric drugs. Nephrology.

[125] Ohtsu N., Anzai N., Fukutomi T., Kimura T., Sakurai H., Endou H. (2010). Human renal urate transpoter URAT1 mediates the transport of salicylate. Nihon Jinzo Gakkai Shi.

[126] Lee M.H., Graham G.G., Williams K.M., Day R.O. (2008). A benefit-risk assessment of benzbromarone in the treatment of gout. Was its withdrawal from the market in the best interest of patients?. Drug Saf..

[127] Persellin R.H., Schmid F.R. (1961). The use of sulfinpyrazone in the treatment of gout. JAMA.

[128] Hamada T., Ichida K., Hosoyamada M., Mizuta E., Yanagihara K., Sonoyama K., Sugihara S., Igawa O., Hosoya T., Ohtahara A. (2008). Uricosuric action of losartan via the inhibition of urate transporter 1 (URAT 1) in hypertensive patients. Am. J. Hypertens..

[129] FDA (2020). In Vitro Drug Interaction Studies—Cytochrome P450 Enzyme- and Transporter-Mediated Drug Interactions Guidance for Industry. https://www.fda.gov/regulatory-information/search-fda-guidance-documents/vitro-drug-interaction-studies-cytochrome-p450-enzyme-and-transporter-mediated-drug-interactions.

[130] Darakhshan S., Pour A.B. (2015). Tranilast: A review of its therapeutic applications. Pharmacol. Res..

[131] Sharpe M., Ormrod D., Jarvis B. (2002). Micronized fenofibrate in dyslipidemia: A focus on plasma high-density lipoprotein cholesterol (HDL-C) levels. Am. J. Cardiovasc. Drugs.

[132] Lee H.A., Yu K.S., Park S.I., Yoon S., Onohara M., Ahn Y., Lee H. (2019). URC102, a potent and selective inhibitor of hURAT1, reduced serum uric acid in healthy volunteers. Rheumatology.

[133] Flamenbaum W., Friedman R. (1982). Pharmacology, therapeutic efficacy, and adverse effects of bumetanide, a new “loop” diuretic. Pharmacotherapy.

[134] Lowe J., Gray J., Henry D.A., Lawson D.H. (1979). Adverse reactions to frusemide in hospital inpatients. Br. Med. J..

[135] Freeman R.B., Duncan G.G. (1960). Chlorothiazide-induced hyperuricemia: Report of two cases. Metab. Clin. Exp..

[136] Aronoff A. (1960). Acute gouty arthritis precipitated by chlorothiazide. N. Engl. J. Med..

[137] Vandell A.G., McDonough C.W., Gong Y., Langaee T.Y., Lucas A.M., Chapman A.B., Gums J.G., Beitelshees A.L., Bailey K.R., Johnson R.J. (2014). Hydrochlorothiazide-induced hyperuricaemia in the pharmacogenomic evaluation of antihypertensive responses study. J. Intern. Med..

[138] Obel A.O., Koech D.K. (1991). Potassium supplementation versus bendrofluazide in mildly to moderately hypertensive Kenyans. J. Cardiovasc. Pharmacol..

[139] Brater D.C. (1996). Benefits and risks of torasemide in congestive heart failure and essential hypertension. Drug Saf..

[140] Wile D. (2012). Diuretics: A review. Ann. Clin. Biochem..

[141] Hamada T., Kuwabara M., Watanabe A., Mizuta E., Ohtahara A., Omodani H., Watanabe M., Nakamura H., Hirota Y., Miyazaki S. (2014). A comparative study on the effectiveness of losartan/hydrochlorothiazide and telmisartan/hydrochlorothiazide in patients with hypertension. Clin. Exp. Hypertens..

[142] Onitsuka H., Koyama S., Ideguchi T., Ishikawa T., Kitamura K., Nagamachi S. (2019). Impact of short-acting loop diuretic doses and cardiac sympathetic nerve abnormalities on outcomes of patients with reduced left ventricular function. Medicine.

[143] Hisatake S., Nanjo S., Fujimoto S., Yamashina S., Yuzawa H., Namiki A., Nakano H., Yamazaki J. (2011). Comparative analysis of the therapeutic effects of long-acting and short-acting loop diuretics in the treatment of chronic heart failure using (123)I-metaiodobenzylguanidine scintigraphy. Eur. J. Heart Fail..

[144] Stevens V.A., Saad S., Poronnik P., Fenton-Lee C.A., Polhill T.S., Pollock C.A. (2008). The role of SGK-1 in angiotensin II-mediated sodium reabsorption in human proximal tubular cells. Nephrol. Dial. Transplant..

[145] Mishima E., Anzai N., Miyazaki M., Abe T. (2020). Uric Acid Elevation by Favipiravir, an Antiviral Drug. Tohoku J. Exp. Med..

[146] Hagos Y., Bahn A., Vormfelde S.V., Brockmoller J., Burckhardt G. (2007). Torasemide transport by organic anion transporters contributes to hyperuricemia. J. Am. Soc. Nephrol..

[147] Uwai Y., Saito H., Hashimoto Y., Inui K.I. (2000). Interaction and transport of thiazide diuretics, loop diuretics, and acetazolamide via rat renal organic anion transporter rOAT1. J. Pharmacol. Exp. Ther..

[148] Hasannejad H., Takeda M., Taki K., Shin H.J., Babu E., Jutabha P., Khamdang S., Aleboyeh M., Onozato M.L., Tojo A. (2004). Interactions of human organic anion transporters with diuretics. J. Pharmacol. Exp. Ther..

[149] Uchida Y., Kamiie J., Ohtsuki S., Terasaki T. (2007). Multichannel liquid chromatography-tandem mass spectrometry cocktail method for comprehensive substrate characterization of multidrug resistance-associated protein 4 transporter. Pharm. Res..

[150] Beery E., Rajnai Z., Abonyi T., Makai I., Bansaghi S., Erdo F., Sziraki I., Heredi-Szabo K., Kis E., Jani M. (2012). ABCG2 modulates chlorothiazide permeability--in vitro-characterization of its interactions. Drug Metab. Pharmacokinet..

[151] Yin J., Wagner D.J., Prasad B., Isoherranen N., Thummel K.E., Wang J. (2019). Renal secretion of hydrochlorothiazide involves organic anion transporter 1/3, organic cation transporter 2, and multidrug and toxin extrusion protein 2-K. Am. J. Physiol. Ren. Physiol..

[152] Vallon V., Rieg T., Ahn S.Y., Wu W., Eraly S.A., Nigam S.K. (2008). Overlapping in vitro and in vivo specificities of the organic anion transporters OAT1 and OAT3 for loop and thiazide diuretics. Am. J. Physiol. Ren. Physiol..

[153] El-Sheikh A.A., Greupink R., Wortelboer H.M., van den Heuvel J.J., Schreurs M., Koenderink J.B., Masereeuw R., Russel F.G. (2013). Interaction of immunosuppressive drugs with human organic anion transporter (OAT) 1 and OAT3, and multidrug resistance-associated protein (MRP) 2 and MRP4. Transl. Res. J. Lab. Clin. Med..

[154] Sekiya M., Matsuda T., Yamamoto Y., Furuta Y., Ohyama M., Murayama Y., Sugano Y., Ohsaki Y., Iwasaki H., Yahagi N. (2020). Deciphering genetic signatures by whole exome sequencing in a case of co-prevalence of severe renal hypouricemia and diabetes with impaired insulin secretion. BMC Med. Genet..

[155] Bhatnagar V., Richard E.L., Wu W., Nievergelt C.M., Lipkowitz M.S., Jeff J., Maihofer A.X., Nigam S.K. (2016). Analysis of ABCG2 and other urate transporters in uric acid homeostasis in chronic kidney disease: Potential role of remote sensing and signaling. Clin. Kidney J..

